# Inferential considerations for low-count RNA-seq transcripts: a case study on the dominant prairie grass *Andropogon gerardii*

**DOI:** 10.1186/s12864-016-2442-7

**Published:** 2016-02-27

**Authors:** Seth Raithel, Loretta Johnson, Matthew Galliart, Sue Brown, Jennifer Shelton, Nicolae Herndon, Nora M. Bello

**Affiliations:** Department of Statistics, Kansas State University, Manhattan, KS USA; Division of Biology, Kansas State University, Manhattan, KS USA

**Keywords:** RNA-seq, Low-count transcripts, *Andropogon gerardii*, DESeq2, EdgeR robust, Gene filtering, Plasmode

## Abstract

**Background:**

Differential expression (DE) analysis of RNA-seq data still poses inferential challenges, such as handling of transcripts characterized by low expression levels. In this study, we use a plasmode-based approach to assess the relative performance of alternative inferential strategies on RNA-seq transcripts, with special emphasis on transcripts characterized by a small number of read counts, so-called low-count transcripts, as motivated by an ecological application in prairie grasses. Big bluestem (*Andropogon gerardii*) is a wide-ranging dominant prairie grass of ecological and agricultural importance to the US Midwest while edaphic subspecies sand bluestem (*A. gerardii ssp. Hallii*) grows exclusively on sand dunes. Relative to big bluestem, sand bluestem exhibits qualitative phenotypic divergence consistent with enhanced drought tolerance, plausibly associated with transcripts of low expression levels. Our dataset consists of RNA-seq read counts for 25,582 transcripts (60 % of which are classified as low-count) collected from leaf tissue of individual plants of big bluestem (*n* = 4) and sand bluestem (*n* = 4). Focused on low-count transcripts, we compare alternative ad-hoc data filtering techniques commonly used in RNA-seq pipelines and assess the inferential performance of recently developed statistical methods for DE analysis, namely DESeq2 and edgeR robust. These methods attempt to overcome the inherently noisy behavior of low-count transcripts by either shrinkage or differential weighting of observations, respectively.

**Results:**

Both DE methods seemed to properly control family-wise type 1 error on low-count transcripts, whereas edgeR robust showed greater power and DESeq2 showed greater precision and accuracy. However, specification of the degree of freedom parameter under edgeR robust had a non-trivial impact on inference and should be handled carefully. When properly specified, both DE methods showed overall promising inferential performance on low-count transcripts, suggesting that ad-hoc data filtering steps at arbitrary expression thresholds may be unnecessary. A note of caution is in order regarding the approximate nature of DE tests under both methods.

**Conclusions:**

Practical recommendations for DE inference are provided when low-count RNA-seq transcripts are of interest, as is the case in the comparison of subspecies of bluestem grasses. Insights from this study may also be relevant to other applications focused on transcripts of low expression levels.

**Electronic supplementary material:**

The online version of this article (doi:10.1186/s12864-016-2442-7) contains supplementary material, which is available to authorized users.

## Background

RNA sequencing (RNA-seq) technology has rapidly become the preferred choice for gene expression analysis as it allows for high throughput over a wide range of expression levels [1]. Yet, some features of RNA-seq data still pose considerable challenges for differential expression (DE) analysis, in particular related to transcripts with low levels of expression, as characterized by low number of read counts [2, 3]. So called low-count transcripts often show large variability of logarithmic fold change (LFC) estimates and thus exhibit inherently noisier inferential behavior [3]. Thus, it is not surprising that standard protocols for processing of RNA-seq data call for filtering out transcripts with read counts below predetermined expression thresholds [4]. As a consequence of data filtering, low-count transcripts are often excluded from DE analyses and ignored for the purpose of inference. Thus, it is plausible that important transcripts of low expression levels, such as transcription factors, may be easily overlooked despite their key role as master regulators of downstream gene expression [5].

Data filtering prior to DE analyses was originally implemented in an attempt to control noise and reduce the impact of multiple testing adjustments on power for DE detection by removal of uninformative or weakly expressed transcripts [4, 6]. Nevertheless, thresholds for filtering are usually specified with little, if any, biological rationale and at seemingly arbitrary cut-offs that vary widely across studies [4, 7, 8]. Recent advances in statistical methods available for DE analyses of RNA-seq data may provide alternative approaches to deal with weakly expressed transcripts without the need for data filtering at arbitrary expression thresholds. More specifically, methods like DESeq2 [3], edgeR [9] and edgeR robust [10] have become particularly popular. Most notably, these methods attempt to handle extremely large counts on RNA-seq transcripts, which may unwittingly also facilitate inference on low-count transcripts. That is, rather than filtering out low-count transcripts at arbitrary cut-off threshold and excluding them from DE analysis, these statistical methods could potentially be used to account for the increased uncertainty associated with low-count transcripts. Both DESeq2 and edgeR have in common a generalized linear mixed models framework that relies on the negative binomial distribution family and efficiently borrows information across transcripts to moderate transcript-specific dispersion estimates [3, 10]. As an additional advantage, DESeq2 shrinks LFC estimates towards a common mean in a manner inversely proportional to the amount of information available for a transcript [3]. Limited information due to either a high level of dispersion (as in extremely large counts) or a low level of expression (as in low-count transcripts) causes transcript-specific LFC estimates to shrink towards zero. In turn, the latest release of edgeR, namely edgeR robust, works by down weighting observations that deviate from the model fit [10], thereby dampening the effect that observations with very high or very low expression levels have on transcript-specific estimates of mean expression and dispersion. As a trade-off, edgeR robust requires specification of a degrees of freedom (DF) parameter that controls the amount of shrinkage in the estimation process. Unless explicitly specified by the user, the DF parameter defaults to a set predetermined value [10] that may be appropriate for some, but not necessarily all data applications. Interestingly, no such user specification is required by DESeq2; rather, all necessary parameters are estimated from the data.

**Fig. 1:**
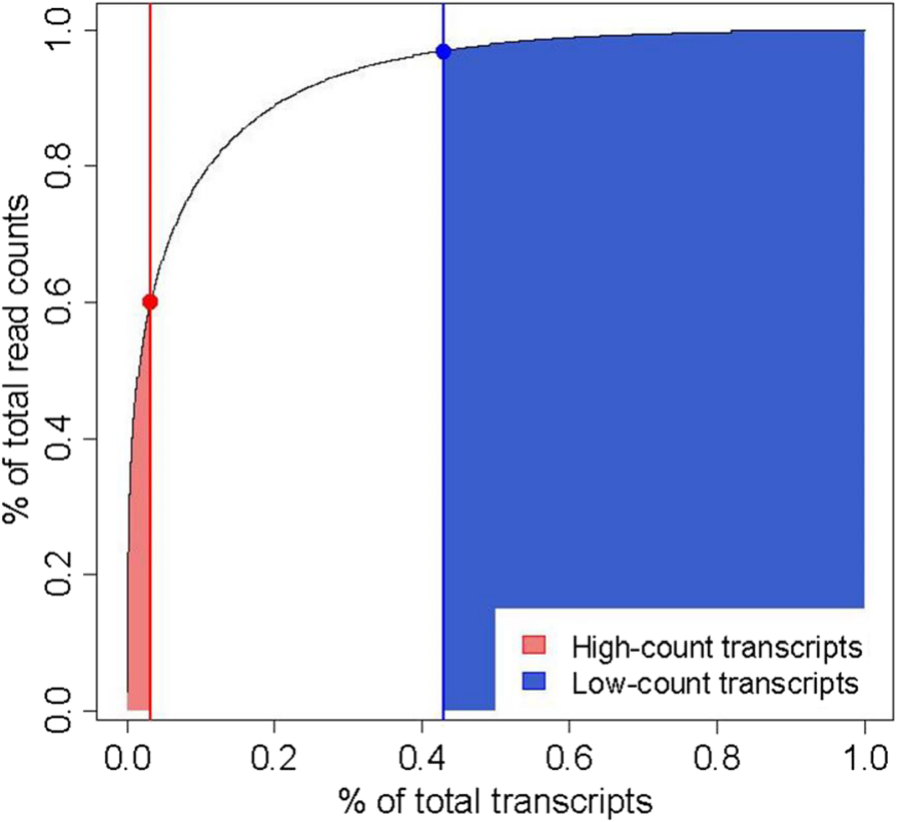
Partitioning of high-count transcripts and low-count transcripts. Cumulative percentage of total read counts (*y*-axis) as a function of cumulative percentage of transcripts (*x*-axis), starting on the left with transcripts of highest read counts. Solid colored lines indicate the 3^rd^ percentile most highly expressed transcripts (*red line*) and the 60^th^ percentile least expressed transcripts (blue line), thereby defining high-count transcripts (*light red to the left of the vertical red line*) and low-count transcripts (*light blue to the right of the vertical blue line*), respectively

In this study, we use a data application on prairie grasses to illustrate inferential challenges related to low-count transcripts in RNA-seq DE analyses. Our motivating interest in low-count RNA-seq transcripts stems from our ongoing work with the wide-ranging prairie grass big bluestem (*Andropogon gerardii*) and its edaphic subspecies sand bluestem (*A. gerardii ssp. Hallii*). Big bluestem (BB) is a widely-distributed dominant grass of North American grasslands [11] and constitutes the main native forage grass for cattle in the US Great Plains [12]. In contrast, sand bluestem’s (SB) habitat consists primarily of the Sand Hills in Nebraska [11–14]. Our preliminary studies [15] pointed towards phenotypic differences between BB and SB subspecies that are consistent with enhanced drought tolerance of SB. For instance, we observed a greater quantity of epicuticular wax on the leaf surface of SB plants relative to that of BB [15]. Further, analysis of epicuticular wax components showed presence of approximately ~20 % β-diketones on SB leaves, whereas β-diketones were absent in epicuticular wax of BB leaves [15]. Differential quantity and quality of epicuticular wax on leaf surfaces could affect heat reflectance and transmittance, thus providing differential relative advantages to heat tolerance in dry conditions. Further, epicuticular wax decreased light absorbance in sand bluestem [15], thus potentially lowering internal leaf temperature and further protecting SB grasses against heat stress. Taken together, our preliminary studies indicate that adaptation of SB to water-limited conditions may involve adaptation of leaf cuticle chemistry, morphology, and function. Sand bluestem’s enhanced tolerance to dry conditions relative to BB [15] is of interest due to the expected increase in extreme droughts throughout Midwest grasslands [16]. In this study, we further characterize differences between BB and SB subspecies at the transcriptome level. Following from the qualitative phenotypic divergence observed between SB and BB, we initially focused on RNA transcripts that were expressed in only one of the bluestem subspecies, with expression absent in the other. More specifically, we considered SB-only transcripts that were expressed in sand bluestem samples but were absent (i.e. counts of 0 reads for all samples) in big bluestem samples and, conversely, BB-only transcripts that were expressed only in BB grasses. We further noticed that these SB-only and BB-only transcripts were characterized by few read counts in the corresponding grasses where they were detected, indicating overall low levels of expression. For this study, we purposely defined so called low-count transcripts following the descriptive approach proposed by Bullard [17], such that low-count transcripts were those below the 60^th ^percentile of least relative abundance, accounting for approximately 3 % of total read counts (Fig. 1). For contrast, we also defined so-called high-count transcripts, corresponding to transcripts in the top 3^rd^ percentile relative abundance and accounting for 60 % of total read counts in the dataset (Fig. 1). Out of 25,582 total transcripts identified in our dataset of bluestem grasses, 14,588 transcripts were defined as low-count transcripts due to a total read count below 462 for each sample (Table 1); whereas 831 transcripts were defined as high-count transcripts with a total read count above 12,893 for each sample (Table 1). Therefore, it is apparent that low-count transcripts constitute a non-trivial subset of transcripts, a substantial part of which would likely be excluded from analyses based on standard data filtering practices. Further, all 132 SB-only transcript and 323 BB-only transcripts in the dataset were also identified as low-count transcripts (Table 1), thus providing specific motivation to our study of transcripts with low expression levels.

**Table 1:** Number of transcripts in the dataset

	All transcripts			High-count transcripts			Low-count transcripts		
	Total	SB-only	BB-only	Total	SB-only	BB-only	Total	SB-only	BB-only
No filter	25,582	323	132	831	0	0	14,588	323	132
RP filtering	25,453	323	132	831	0	0	14,588	323	132
CPM filtering	14,848	0	0	828	0	0	4308	0	0

The table contains the number of total transcripts, high-count transcripts and low-count transcripts available for differential expression analyses in the complete dataset (i.e. no data filtering applied) or following data filtering based on a reads-present (RP) criterion or a counts per million (CPM) criterion. Also listed are number of transcripts with expression levels present in sand bluestem and absent in big bluestem (SB-only transcripts), and transcripts with expression levels present in big bluestem and absent in sand bluestem (BB-only transcripts)

To deal with the additional uncertainty that characterizes low-count transcripts due to limited information in their low level of expression, we implement the recently developed statistical methods edgeR robust and DESeq2, both having shown promising results in simulation studies and in selected real datasets [3, 10]. However, relative performance of these methods has often been shown to be data dependent, such that their relative results may differ across datasets [10]. Thus, it is unclear how one might decide between these statistical methods for DE analysis in a specific situation. Plasmodes have been proposed as a strategy to validate statistical methods, or even assess relative performance of competing methods, on a given -omics dataset [18]. Probably one of the main advantages of plasmodes is that some characteristics of a particular dataset of interest can be preserved when assessing inference, including the overall distribution of the data as well as any potential gene-to-gene correlation structure [7]. More specifically, an RNA-Seq dataset can be used to generate a null plasmode dataset by randomly partitioning samples of the same treatment condition into two new arbitrary groups. Reshuffling of samples creates a null dataset for which no differential expression between groups is to be expected beyond sample-to-sample variation [7]. Null plasmodes may then be subjected to DE analysis to compare Type I error between methods, as any transcripts identified as DE on a null plasmode would be considered a false positive. Plasmodes also allow for the introduction of some known truth, as it happens with simulated data, whereby selected transcripts in a plasmode can be “spiked” with fold changes of known magnitude to create DE transcripts, and thus evaluate statistical power and other performance metrics under the alternative hypothesis [7]. Thus far, plasmodes have been successfully applied to microarray [19] and qPCR data [20], and have most recently been adapted for RNA-Seq data as well [7].

In this study, we use a plasmode-based approach to assess inferential performance of statistical methods for DE analysis of RNA-seq data with a special focus on low-count transcripts, as illustrated by our case study on bluestem prairie grasses. In particular, we evaluate the inferential performance of DESeq2 and edgeR, with variations on the specification of the DF parameter beyond default values for the latter method. We further evaluate the impact of data filtering strategies that are commonly reported in the literature and discuss their implications for inference on low-count transcripts. We believe it is timely that guidelines for data filtering of low expression transcripts in protocols for RNA-seq data [4] are revisited, as the impact of this practice on DE inference remains unclear, particularly for transcripts that may be biologically relevant despite low expression levels (e.g. transcription factors).

## Results

### Plasmodes

Given the benchmark status of BB as a widely distributed dominant prairie grass, we generated all plasmodes using BB samples only. To evaluate inferential performance of statistical methods under the null hypothesis, samples of individual BB plants (*n* = 4) were randomly partitioned into 2 groups of 2 samples each, yielding a total of 3 null plasmodes. In turn, performance under the alternative hypothesis was evaluated using DE plasmodes, that is, modified null plasmodes for which one of the groups had a known proportion of transcripts “spiked” with estimates of effect sizes of transcripts called DE from a preliminary analysis [7]. A total of 15 DE plasmodes were generated using all 3 null plasmodes as baseline.

On each plasmode, we conducted DE analyses using DESeq2, edgeR classic, and edgeR robust. All of these statistical methods model read counts assuming a negative binomial conditional data likelihood distribution and apply shrinkage to moderate the estimation of dispersion parameters. For edgeR robust, we specified the DF parameter to take values 4, 10 or 50, thereby reflecting increasing levels of arbitrarily specified shrinkage. We note that DF = 10 is the default DF specification in edgeR robust, unless otherwise specified by the user. We also evaluated the performance of edgeR robust with DF specified using an estimate obtained from the classic edgeR software (i.e. $\mathrm{D}\mathrm{F} = {\widehat{DF}}_{edgeR}$). We note that a quantile-adjusted conditional maximum likelihood approach for estimation of the DF parameter is available in the classic edgeR software for simple, completely randomized design structures such as that in our motivating problem on bluestem subspecies [21]. Estimated DF values ranged from ${\widehat{DF}}_{edgeR}=3.21\ \mathrm{t}\mathrm{o}\ 3.30$ across null plasmodes, which is noted to be considerably smaller than the default specification (i.e. DF = 10). To compare performance of the various DE analyses methods, we computed false positive rate (FPR), true positive rate (TPR) or power, positive predictive value (PPV) or precision, negative predictive value (NPV), and accuracy, as defined in Table 2.

**Table 2:** Classification rules to compute performance metrics

	Transcripts not DE	Transcripts “spiked” for DE	Total
Transcripts not declared significantly DE	TN	FN	R_0_
Transcripts declared significantly DE	FP	TP	R_1_
Total	S_0_	S_1_	G

*FP* number of false positives (transcripts in S_0_ set declared differentially expressed); *TP* number of true positives (transcripts in S_1_ set declared differentially expressed); *TN* number of true negatives; *FN* number of false negatives; FPR, false positive rate = FP/S_0_; *TPR* true positive rate or power = TP/S_1_; *PPV* positive predictive value or precision = TP/R_1_; *NPV* negative predictive value = TN/R_0_; accuracy = (TP + TN)/G

We first assessed Type I error of DE methods on null plasmodes using FPR. Since both groups in the null plasmode pertain to the same subspecies and are arbitrarily defined, we do not expect any group differences in expression levels beyond sampling variability. Table 3 contains estimated FPR for the DE methods implemented here, after adjustment to a false discovery rate (FDR) of 0.05. Overall, all methods seemed to adequately control FPR below a 0.05 FDR nominal value for both all transcripts as well as low-count transcripts. Nevertheless, DESeq2 had the lowest FPR and was thus the most conservative of the methods evaluated, followed closely by edgeR classic and then by edgeR robust. Within edgeR robust, FPR increased with more degrees of freedom, thus indicating more liberal inference with greater DF specifications, though in all cases within the nominal 0.05 value. These patterns in FPR performance between DE methods were apparent when either all transcripts or only low-count transcripts were considered.

**Table 3:** Estimated false positive rates (FPR) on null plasmodes

edgeR robust DF = 50	edgeR robust DF = 10	edgeR robust DF = 4	edgeR robust $\mathrm{D}\mathrm{F} = \widehat{DF}$	edgeR classic $\mathrm{D}\mathrm{F} = \widehat{DF}$	DESEQ2
FPR - All transcripts					
0.0177 a	0.0093 b	0.0063 c	0.0061 c	0.0042 d	0.0031 e
(0.00040)	(0.00040)	(0.00040)	(0.00040)	(0.00040)	(0.00040)
FPR - Low-count transcripts					
0.0176 a	0.0094 b	0.0064 c	0.0063 c	0.0043 d	0.0032 e
(0.00037)	(0.00037)	(0.00037)	(0.00037)	(0.00037)	(0.00037)

Least square mean estimates (and corresponding SEM, shown in parentheses) of FPR for differential expression at FDR = 0.05 on all transcripts and on low-count transcripts based on DESeq2, EdgeR classic and EdgeR robust, implemented on null plasmodes of RNA-seq data. a,b,c,d,e, indicate significant differences (Tukey-Kramer adjusted *P* < 0.05) within a row

Next, we used DE plasmodes to compare inferential performance of statistical methods under the alternative hypothesis to detect true differences in expression levels of transcripts. Estimated TPR or power, PPV or precision, NPV and accuracy based on DE plasmodes are displayed in Table 4. Estimated power across methods ranged from approximately 0.54 to 0.65 for all transcripts, and from 0.17 to 0.39 for low-count transcripts. In both cases, DESeq2 showed the lowest power, followed by a modest power increase with edgeR classic and a more substantial power boost with edgeR robust. Within specifications of edgeR robust, there was no evidence for differences in power when DF were specified to be 10 or less, but a DF = 50 specification caused a significant increase in power both for all transcripts and for low-count transcripts. Not surprisingly, results on power mirrored those obtained on FPR based on the null plasmodes; that is, methods with the lowest FPR were also methods with the highest number of false negatives and thus, the lowest power. This can be explained by a well-known trade-off between Type I and Type II errors in statistical inference.

**Table 4:** Performance metrics on differentially expressed (DE) plasmodes

edgeR robust DF = 50	edgeR robust DF = 10	edgeR robust DF = 4	edgeR robust $\mathrm{D}\mathrm{F} = \widehat{DF}$	edgeR classic $\mathrm{D}\mathrm{F} = \widehat{DF}$	DESEQ2
Power - All transcripts					
0.6495 a	0.6275 b	0.6215 b	0.6229 b	0.5704 c	0.5418 d
(0.00610)	(0.00610)	(0.00463)	(0.00463)	(0.00435)	(0.00435)
Power - Low-count transcripts					
0.3922 a	0.3692 b	0.3657 b	0.3677 b	0.2647 c	0.1785 d
(0.00120)	(0.00120)	(0.00010)	(0.00010)	(0.00008)	(0.00008)
Precision - All transcripts					
0.3607 a	0.4393 b	0.5218 c	0.5323 c	0.6321 d	0.6586 e
(0.00792)	(0.01153)	(0.01192)	(0.01114)	(0.00589)	(0.00371)
Precision - Low-count transcripts					
0.1800 a	0.2289 b	0.2904 c	0.2963 c	0.3605 d	0.3915 e
(0.00811)	(0.01305)	(0.01432)	(0.01378)	(0.01363)	(0.01727)
NPV - All transcripts					
0.9917 a	0.9915 a	0.9915 a	0.9915 a	0.9902 b	0.9891 c
(0.00016)	(0.00016)	(0.00016)	(0.00016)	(0.00016)	(0.00016)
NPV - Low-count transcripts					
0.9917 a	0.9915 a	0.9914 a	0.9915 a	0.9902 b	0.9891 c
(0.00016)	(0.00016)	(0.00016)	(0.00016)	(0.00016)	(0.00016)
Accuracy - All transcripts					
0.9718 a	0.9778 b	0.9821 b	0.9826 c	0.9858 d	0.9862 e
(0.00080)	(0.00080)	(0.00024)	(0.00024)	(0.00012)	(0.00014)
Accuracy - Low-count transcripts					
0.9679 a	0.9744 b	0.9794 c	0.9798 c	0.9841 d	0.9855 e
(0.00122)	(0.00122)	(0.00079)	(0.00079)	(0.00028)	(0.00028)
FPR - All transcripts					
0.0221 a	0.0155 b	0.011 b	0.0106 c	0.0063 d	0.0053 e
(0.00086)	(0.00086)	(0.00024)	(0.00024)	(0.00001)	(0.00010)
FPR - Low-count transcripts					
0.0244 a	0.0175 b	0.0124 c	0.0121 c	0.0063 d	0.0037 e
(0.00128)	(0.00128)	(0.00082)	(0.00082)	(0.00019)	(0.00019)

Least square mean estimates (and corresponding SEM, shown in parentheses) for true positive rate (TPR; i.e. power), positive predictive value (PPV; i.e. precision), negative predictive value (NPV), accuracy and false positive rate (FPR) for differential expression at FDR = 0.05 on all transcripts and on low-count transcripts yielded by DESeq2, EdgeR classic or EdgeR robust, implemented on DE plasmodes of RNA-seq data. a,b,c,d,e, indicate significant differences (Tukey-Kramer adjusted *P* < 0.05) within a row

Precision, or PPV, was maximum using DESeq2 and was estimated at 0.66 and 0.39 for all transcripts and low-count transcripts, respectively (Table 4). In both cases, a significant drop in precision of at least 2 to 3 percentage points was apparent with edgeR classic relative to DESeq2, whereas the estimated drop in precision was of 10 percentage points or more with edgeR robust relative to DESeq2. As the specification of DF on edgeR robust increased from 4 to 50, inferential precision decreased further and was nearly halved using edgeR robust with DF = 50 relative to DESeq2. Noteworthy, both for all transcripts and for low-count transcripts, inferential precision using edgeR robust was greater by approximately 7 to 11 percentage points when DF were estimated as opposed to specified by default (i.e. DF = 10; Table 4). In turn, estimated NPV for all DE methods was high in magnitude and ranged from 0.989 to 0.992 for all transcripts as well as for low-count transcripts (Table 4).

Overall inferential accuracy of DE analyses ranged from 0.972 to 0.986 for all transcripts and from 0.968 to 0.985 for low-count transcripts. In both cases, maximum accuracy was observed using DESeq2, followed in decreasing order by edgeR classic and then by edgeR robust, with decreasing accuracy as DF increased (Table 4). Again, overall accuracy of DE calling using edgeR robust was greater when DF were estimated as opposed to specified by default (i.e. DF = 10), though the absolute magnitude of the difference was small (approximately one percentage point). All methods appeared to control FPR in DE plasmodes below the nominal value (Table 4), though DESeq2 was more conservative than any of the edgeR methods, particularly for low-count transcripts.

### Case study: comparison of bluestem subspecies

**Fig. 2:**
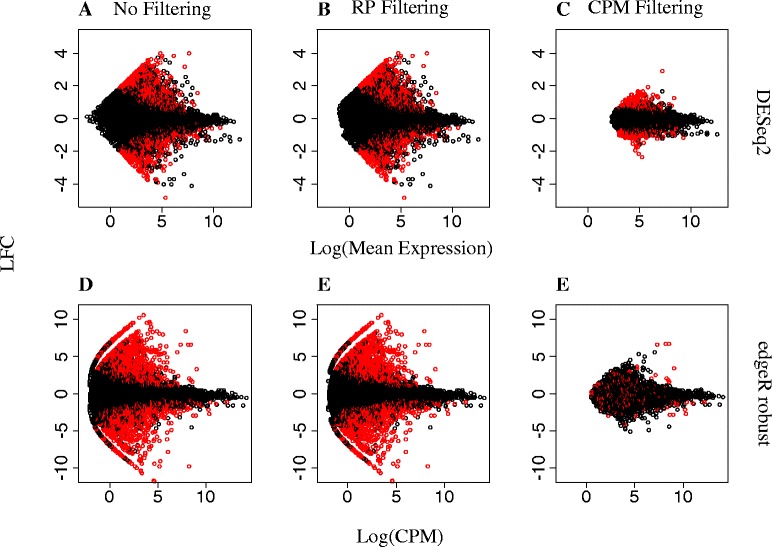
MA-Plots for edgeR robust and DESeq2 with and without data filtering. Estimated fold-change in expression of RNA-seq transcripts for SB relative to BB as a function of transcript abundance following differential expression analyses with DESeq2 or edgeR robust (DF = Classic $\widehat{\mathrm{D}}\mathrm{F}$=_edgeR) on data subjected to no filtering (**a**, **d**) or to filtering with CPM (**c**, **f**) or RP (**b**, **e**) methods. For DESeq2, fold-changes are plotted over mean transcript expression on a log scale. For edgeR robust, fold-changes are plotted against counts per million on a log scale. Transcripts declared DE at FDR = 0.05 are colored in red

**Fig. 3:**
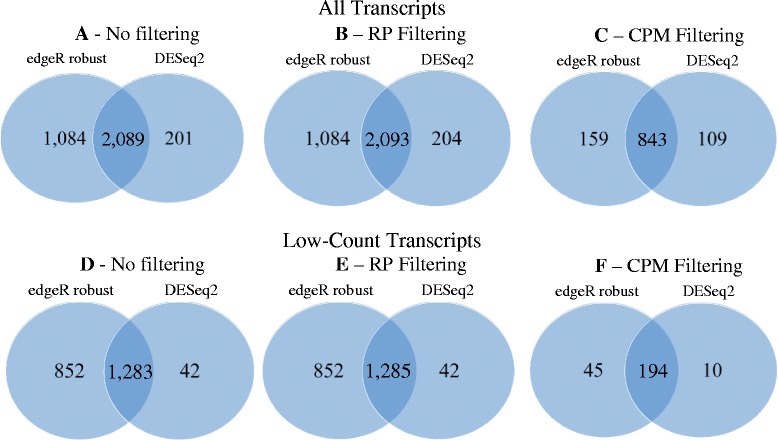
Frequency of transcripts declared differentially expressed (DE) using edgeR robust and DESeq2. Venn diagrams of all transcripts and of low-count transcripts declared DE using edgeR robust (with degrees of freedom specified based on the corresponding estimate obtained using classical edgeR software) and DESeq2 on the complete dataset (i.e. no data filtering) or following data filtering based on a reads-present (RP) criterion or a counts per million (CPM) criterion

Next, we conducted illustrative DE analyses to explore the transcriptomic basis for differences between BB and SB subspecies of bluestem prairie grass, with emphasis on SB-only and BB-only transcripts, all of which were characterized by low levels of expression. Our dataset consisted of 4 samples of big bluestem and 4 of sand bluestem, for which read counts on a total of 25,582 transcripts were obtained. Differential expression analyses between subspecies were conducted using DESeq2 and edgeR robust. The specification of DF for edgeR robust was based on quantile-adjusted conditional maximum likelihood estimates using edgeR classic [21], whereby ${\widehat{DF}}_{edgeR}=3.02.$ Figure 2a and d contain MA plots of estimated logarithmic fold changes in the complete dataset (i.e. no filtering) using DESeq2 and edgeR robust, respectively. Overall, edgeR robust declared 12.4 % (3173 out of 25,582) of transcripts as DE (Table 5) whereas DESeq2 declared only 9.0 % (2290 out of 25,582) transcripts as DE (Table 6). This is consistent with results from our plasmode study in the previous section, whereby DESeq2 had a more conservative Type I error performance relative to edgeR robust, coupled with greater power of the latter. Differences in DE calling between statistical methods was explained, at least partially, by low-count transcripts, whereby 14.6 % (2135 out of 14,588) of low-count transcripts were declared DE by edgeR robust but only 9.1 % (1325 out of 14,588) were by DESeq2 (Tables 5 and 6). Instead, DE calling amongst high-count transcripts was 4.8 % (40 out of 831) and 4.6 % (38 out of 831) for edgeR robust and DESeq2, respectively (Tables 5 and 6). Overall, a considerable amount of overlap in DE calling was apparent between methods, as approximately 91.2 % of all transcripts declared DE by DESeq2 were also declared DE using edgeR robust (Fig. 3a). For low-count transcripts in the complete dataset, edgeR robust declared DE approximately 96.8 % of those also declared DE by DESeq2 (Fig. 3d).

**Table 5:** Number of transcripts declared differentially expressed (DE) using edgeR robust

	All transcripts			High-count transcripts	Low-count transcripts		
	Total	SB-only	BB-only	Total	Total	SB-only	BB-only
No filter	3173	248	126	40	2135	245	121
RP filtering	3177	248	126	40	2137	245	121
CPM filtering	1002	0	0	23	239	0	0

The table contains the number of total transcripts, high-count transcripts and low-count transcripts declared DE using edgeR robust (with degrees of freedom specified based on the corresponding estimate obtained using classical edgeR software) analyses on the complete dataset (i.e. no data filtering applied) or following data filtering based on reads-present (RP) criterion or counts per million (CPM) criterion. Also listed are transcripts with expression levels present in sand bluestem and absent in big bluestem (SB-only transcripts) and transcripts with expression levels present in big bluestem and absent in sand bluestem (BB-only transcripts)

**Table 6:** Number of transcripts declared differentially expressed (DE) using DESeq2

	All transcripts			High-count transcripts	Low-count transcripts		
	Total	SB-only	BB-only	Total	Total	SB-only	BB-only
No filter	2290	112	69	38	1325	112	69
RP filtering	2297	111	69	38	1327	111	69
CPM filtering	952	0	0	30	204	0	0

The table contains the number of total transcripts, high-count transcripts and low-count transcripts declared DE using DESeq2 analyses on the complete dataset (i.e. no data filtering applied) or following data filtering based on reads-present (RP) criterion or counts per million (CPM) criterion. Also listed are transcripts with expression levels present in sand bluestem and absent in big bluestem (SB-only transcripts) and transcripts with expression levels present in big bluestem and absent in sand bluestem (BB-only transcripts)

We further considered SB-only and BB-only transcripts expressed in only one bluestem subspecies and absent in the other. Recall that all such transcripts were classified as low-count transcripts due to low expression levels. EdgeR robust identified 80.4 % (i.e. (245 + 121)/(323 + 132)) of such transcripts as DE (Table 5), whereas DESeq2 called DE only 39.8 % (i.e.(112 + 69)/(323 + 132)) (Table 6). Yet, approximately 99 % of transcripts expressed in only one bluestem subspecies and declared DE based on DESeq2 were also declared DE by edgeR robust, again indicating a substantial amount of overlap between the methods.

### Filtering strategies

We further assessed inferential implications of two commonly used filtering approaches on RNA-seq data. Samples of BB and SB were subjected to filtering of transcripts defined in terms of mapped reads present (RP) [8] and of read counts per million (CPM) [7]. Recall that any specific data filtering approach determines the transcriptomic basis to which DE analyses are later applied, and thus, any DE results.

Criteria for data filtering varies across strategies. Specifically, the RP filtering approach indicates removal of a transcript if the overall number of samples with mapped reads present for that transcript (i.e. samples with read counts greater than zero) is smaller than the number of samples per treatment group (i.e. 4 in this case) [8]. In turn, CPM-based filtering indicates removal of a transcript if a pre-selected number of samples have read CPM for that transcript that are smaller than a pre-selected threshold value [7], specified at 1 CPM for this study. Table 1 shows a breakdown of transcripts available for DE analyses after RP-based filtering and CPM-based filtering. Most notably, RP-based filtering excluded only 129 transcripts (i.e. approximately 0.5 %) from the unfiltered dataset, none of which were low-count transcripts or transcripts present in only one of the subspecies. In contrast, when CPM-based filtering was implemented, a total of 10,734 transcripts (i.e. almost 42 % of the total) were excluded from the data, amongst which were 10,280 low-count transcripts as well as all BB-only transcripts and all SB-only transcripts (Table 1). As such, only approximately 29 % low-count transcripts, and none of the transcripts present in only one of the bluestem subspecies, were available for DE analyses following CPM-based filtering.

Next, filtered datasets were subjected to DE analysis using edgeR robust and DESeq2, as described in the previous section. Tables 5 and 6 show the breakdown of transcripts declared DE by each of the statistical methods on the filtered datasets. Transcripts declared DE in RP-filtered data were essentially the same transcripts declared DE in the unfiltered data (i.e. over 99 % overlap) regardless of DE analyses. Exceptions included additional 4 transcripts (with edgeR robust) or 7 transcripts (using DESeq2) declared DE in the RP-filtered data, but not in unfiltered data. Instead, CPM filtering reduced the number of transcripts declared DE based on edgeR robust by 68.4 % (that is ((3173-1002)/3173), Table 5) and based on DESeq2 by 58.4 % (that is ((2290-952)/2290), Table 6), respectively, relative to unfiltered data. The impact of CPM filtering on DE calling was primarily driven by low-count transcripts, for which DE calling was reduced by approximately 88.8 % (that is ((2135-239)/2135), Table 5) based on edgeR robust and by 84.6 % (that is ((1325-204)/1325), Table 6) based on DESeq2. Most notably, all 455 transcripts present in only one of the bluestem subspecies, that is BB-only and SB-only transcripts, were lost to DE inference as CPM-based filtering excluded them from the data prior to DE analyses.

Figure 2 shows MA-plots obtained from fitting DESeq2 or edgeR robust to RNA-seq data subjected to no filtering (2.A and 2.D), RP-based filtering (2.B and 2.E) or CPM-based filtering (2.C and 2.F) . Within each DE method, the overall shape of the MA-plots on RP-filtered data resembled that of the unfiltered data. This is not surprising as RP filtering removed only a small proportion (approximately 0.5 %) of transcripts from the dataset. In contrast, MA-plots on the CPM-filtered dataset showed a drastically modified pattern relative to unfiltered data, particularly on the left side of each plot, due to a disproportionate exclusion of low-count transcripts, which were also transcripts of more extreme fold-change estimates. Within each filtering strategy, the overlap in DE calling by edgeR robust relative to DESeq2 ranged from 88 to 91 % and from 95 to 97 % for all transcripts and for low-count transcripts, respectively (Fig. 3b, c, e, f).

### Approximate tests for DE inference based on DESeq2

The most recent release of the DESeq package, namely DESeq2 [3], implemented a Wald test approach as the default strategy for DE testing on individual transcripts. This approach differs from that of previous versions of DESeq, which instead specified by default a likelihood ratio test (LRT) [22]. The rationale behind moving towards a Wald test as the default approach seemed to rely on its flexibility for testing individual coefficients or functions thereof, without the need to fit a reduced model [3]. Yet, one should recognize the approximate nature of both tests, which relies on large sample approximations and assumes either an asymptotic chi-square distribution (LRT) or a normal distribution (Wald test) under the null hypothesis [23].

**Fig. 4:**
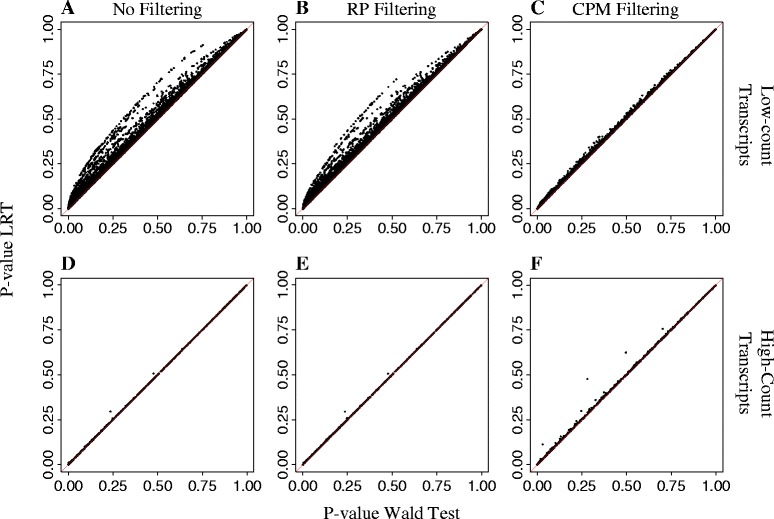
Comparison of P-values for DESeq2 tests on differential expression. Scatterplot of P-values for differential expression obtained using DESeq2’s likelihood ratio test (LRT) and Wald test on low-count and high-count transcripts subjected to no filtering (**a**, **d**) or to filtering with CPM (**c**, **f**) or RP (**b**, **e**) methods. Diagonal identity line is indicated in red

Motivated by our interest in low-count transcripts, we further compared the relative performance of DESeq2-based LRT and Wald tests for DE inference on individual transcripts. Figure 4 shows scatterplots of unadjusted P-values for DE inference obtained from Wald tests (x-axis) and LRT (y-axis) for both high-count transcripts and low-count transcripts based on a complete dataset (i.e. no filtering applied) and on filtered datasets. For high-count transcripts, LRT and Wald tests showed considerable inferential agreement for DE calling regardless of data filtering, as indicated by most points falling along the identity line (Fig. 3d, e, f). In contrast, for low-count transcripts, the Wald test had lower P-values for DE inference relative to the LRT approach (Fig. 3a, b, c). For this application, it is unclear if the Wald test underestimated these P-values or if the LRT overestimated them. This discrepancy is particularly concerning for transcripts with P-values of small magnitude and close to a pre-specified significance threshold given the qualitative differences in inference (i.e. a transcript is either called DE or not). This difference in inferential performance observed between Wald test-based and LRT-based P-values for low-count transcripts was particularly noticeable in the complete dataset (i.e. no filtering applied) and on the RP-filtered data, which included all low-count transcripts. Instead, P-values from Wald test and LRT were more closely aligned to each other on CPM-filtered data. This was expected as most low-count transcripts had already been excluded from DE analyses due to being removed during CPM filtering.

## Discussion

In this study, we used plasmodes generated from RNA-seq data to compare inferential performance of statistical methods for differential expression, with a special focus on low-count transcripts, as motivated by an application on bluestem prairie grasses. More specifically, we evaluated the inferential performance of the recently developed statistical methods DESeq2, edgeR and edgeR robust, the latter specified over a range of the DF parameter. We also considered data filtering strategies that, while pervasively implemented in RNA-seq data pipelines, impose arbitrary criteria for data exclusion, the impact of which on DE inference was shown to be substantial.

This study is one of few to use a plasmode-based approach to compare statistical methods on a specific RNA-seq dataset and more specifically, on a subset of transcripts characterized by low expression levels [7]. We recognize, though, that our null plasmode approach can be sensitive to limited random partitioning of samples into groups, especially if the number of samples available is small, as is our case [19]. By design, though, plasmodes allow for evaluation of inferential performance while taking into consideration the realistic conditions of a given data application [19], amongst which one may consider the actual structure of a real dataset and a limited sample size.

Our results on inferential performance indicated adequate control of Type I error within nominal levels using either DESeq2 or edgeR robust both for all transcripts and for low-count transcripts. Still, false positive rates increased with greater degrees of freedom under edgeR robust, indicating the need for careful consideration of how this parameter is specified. In turn, edgeR robust showed greater power than DESeq2, which is consistent with results from previous simulation studies [10] and is hereby shown to also apply to transcripts of low expression levels. Interestingly, within specifications of edgeR robust, there was no evidence for any changes in power when DF were specified to be at default (i.e. DF = 10) or at a much smaller value estimated from the data (i.e. DF = [3.21, 3.30], though power was significantly inflated with DF = 50. Yet, the observed increase in power with increasing DF in edgeR robust was counterbalanced by an even greater increase in false positives, which in turn impaired inferential precision. Not unexpectedly, power for DE calling of low-count transcripts was decreased relative to that of all transcripts, regardless of method chosen for DE inference. This is to be expected as low levels of expression indicate little information available for inference on a given transcript, as shown by previous simulation studies [10]. Furthermore, DESeq2 showed the greatest precision and accuracy of all methods evaluated not only for all transcripts, as already shown by other simulation studies [3], but especially for low-count transcripts.

Our results from the plasmode-based approach to assess inferential performance suggest that the specification of DF for edgeR robust can impact DE inference of RNA-seq data, particularly that of low-count transcripts and thus, should be considered carefully. Most relevant to our dataset, the default DF specification (i.e. DF = 10) was not optimal and led to a decrease in inferential precision and accuracy relative to using an estimated DF value. The default value of the shrinkage parameter for edgeR robust (i.e. DF = 10) seems to be based on an assortment of simulation studies [9]. However, it is unclear whether such an arbitrarily specified DF value is justified for any particular real RNA-seq dataset, for which the amount of dispersion, the correlation structure between transcripts and the sample size may not be aligned with those of simulated conditions [7, 8]. Our results indicate that the specification of DF on edgeR robust should be informed carefully. This is consistent with warnings raised in other areas of genomic applications about the arbitrary specification of low-level hyperparameters in hierarchical models [24, 25]. It would be recommended for DF to be estimated from the data whenever possible. Alternatively, if complexity of the experimental design prevented proper estimation of the DF parameter, a researcher might consider relying more heavily on inference from DESeq2, for which no arbitrary specification of DF is needed. This is consistent with the relatively standard recommendation that DE inference be based on multiple analysis methods [1, 8]. For instance, researchers may consider declaring DE only those transcripts that show low FDR-adjusted P-values by both DESeq2 and edgeR robust with properly specified degrees of freedom. This recommendation is further supported by the high level of overlap in DE calling observed between the methods, when properly specified.

Data filtering is a common processing step in the RNA-seq data management pipeline [4], though its implications have not been thoroughly explored. Initial implementations of data filtering for RNA-seq counts were intended to reduce the impact of multiple testing adjustment on power for DE detection [4, 6]. Yet, one should note that filtering strategies apply rather arbitrary data exclusion criteria with different, potentially disproportionate, consequences for inference on specific transcripts. For example, our results indicate only a very minor difference in DE calling following RP-based filtering compared to no filtering, with 99 % overlap between the two, regardless of DE method. This suggests that both edgeR robust and DESeq2 retained similar transcripts declared as DE regardless of whether the data has been RP-filtered or not. This result thus questions the very need to impose arbitrary filtering rules on the data given the powerful statistical methods available. In turn, more extreme filtering rules such as those based on a CPM criterion caused a drastic reduction in the transcriptomic basis that was made available for DE analyses. In our case, CPM filtering excluded almost 42 % of the original transcripts, most of which were low-count transcripts. Filtering by CPM criterion was originally designed to remove transcripts considered challenging for inference due to shortage of available information [4]. However, we showed that CPM-based filtering also excluded from the data all transcripts expressed in only one of the bluestem subspecies and absent in the other (i.e. SS-only and BB-only transcripts), which were of particular interest to researchers in the motivating data application. Filtering out these transcripts excludes them from any follow-up DE consideration, which may in turn impair understanding of the transcriptomic basis for phenotypic differences between bluestem subspecies and misinform further exploration of candidate genes. Moreover, CPM-based filtering also reduced both the total number and the proportion of transcripts called DE relative to no filtering, whereas little gain was obtained in uniquely identified DE-declared transcripts (i.e. approximately 0.3 % and 0.1 % gain with DESeq2 and edgeR robust, respectively). Taken together, our results indicate that the implications of data filtering for DE inference should not be taken lightly, as the effect seems to depend, and disproportionally so, on the specifics of the data exclusion criterion as well as on the types of transcript of interest. On a more general note, data exclusion based on CPM filtering may have even more serious implications for inference on transcription factors, which have low expression levels despite their key role as master switches that regulate gene expression [5].

Overall, the rationale for arbitrary filtering RNA-seq data based on either a RP criterion or a CPM criterion seems poorly justified, either biologically or otherwise, particularly given the availability of powerful state-of-the-art statistical methodology developed to deal with the associated challenges in RNA-seq data. Instead, researchers may consider using the complete unfiltered RNA-seq data for DE analyses, ensuring use of modern statistical methods to properly borrow information across transcripts and moderate (i.e. shrink or weigh) DE inference based on expression levels. In particular, DESeq2 and edgeR robust have shown promising inferential performance in handling low-count transcripts with minimal effect on the DE analysis for the remaining transcripts. Further, forgoing the use of data filtering at arbitrary thresholds in favor of more elegant approaches to deal with the inherent challenges of RNA-seq data may be particularly relevant for research questions focused on biologically relevant transcripts characterized by low expression levels, such as transcription factors.

Finally, it is of concern that differential expression assessments for low-count transcripts based on the Wald test implemented by default in DESeq2 yielded more liberal results relative to those based on a likelihood ratio test. Both tests assume that certain regularity conditions hold, though such conditions are rarely verified in practice [23]. It is further concerning that the performance of these approximate tests is known to deteriorate rapidly in situations of limited information, and apparently more so for Wald tests [26]. Both tests implemented by DESeq2 constitute approximations that may require careful attention and detailed consideration of the assumptions made on a case-by-case basis, in order to ensure sound inference and prevent inflation of Type I error.

## Conclusions

We implemented a recently adapted plasmode-based approach to compare inferential performance of modern statistical methods, namely DESeq2 and edgeR robust, on RNA-seq data. Motivated by interest on a transcriptomic comparison of bluestem grass species, we pay special attention to transcripts of low expression levels, defined here as low-count transcripts. We emphasize that implications of these results may be relevant to other biological applications that involve transcripts of high biological importance but low expression levels, such as transcription factors. Both DESeq2 and edgeR robust seemed to properly control family-wise type 1 error on all transcripts as well as on low-count transcripts. For low-count transcripts, edgeR robust showed greater power whereas DESeq2 showed greater precision and accuracy. Overall, both methods showed promising inferential performance on low-count transcripts and yielded a substantial amount of overlap in DE calling, thus supporting their combined use for fine-tuned DE inference. Still, a note of caution is in order regarding the approximate nature of DE tests on individual transcripts, particularly low-count ones.

Regarding edgeR robust, the specification of a degree of freedom parameter was found to be non-trivial. This is to be expected as degrees of freedom determine the amount of shrinkage and borrowing of information across transcripts, thereby impacting precision and accuracy of DE inference. Our results raise legitimate questions about the use of a default value for the degree of freedom hyperparameter, recognizing that a default value may not be appropriate for all datasets, as it was certainly not optimal in our case study. The edgeR robust degrees of freedom parameter should thus be given careful consideration in any data application and, whenever possible, it should be estimated from the data.

Finally, our results support that filtering of RNA-seq data can have serious implications for inference as mostly low-count transcripts are removed from the data and excluded from DE analyses. Standard RNA-seq data management pipelines that call for filtering transcripts out at arbitrary thresholds should be reconsidered. Instead, researchers may implement modern state-of-the-art statistical methodologies specifically developed to deal directly with the inherent challenges of RNA-seq data, including transcripts of low expression levels.

## Methods

### Data collection

RNA was extracted from leaf tissue of 4 individual plants of each of two phenotypically divergent bluestem subspecies, namely big bluestem (*Andropogon gerardii,* Saline population) and sand bluestem (*A. gerardii ssp. Hallii,* Arapahoe population). Phenotypic divergence was established using a species specific, established hybrid index [27]. All plants were grown in common soil under greenhouse conditions. Samples were sequenced using Roche 454 pyrosequencer and Illumina HiSeq 2000 sequencer sequencers. To ensure deeper coverage, one sand bluestem plant (Araphaoe population) and one big bluestem plant (Saline population) were used for analysis on the 454 sequencer. For the 454 run, we used a full plate divided equally between the sand bluestem and big bluestem subspecies. This run yielded 616,333 high quality reads for sand bluestem and 534,633 for big bluestem (total 1,150,966; Additional file 1: Table S1). For the Ilumina HiSeq run, we used 4 biological replicates of the sand bluestem Arapahoe population and 4 biological replicates of the big bluestem Saline population; there were a total of 203,506,904 quality reads for sand bluestem and 172,519,460 high quality reads for big bluestem (total combined 376,026,364; Additional file 1: Table S1). Reads were mapped to a *de-novo* reference transcriptome assembly (described next) [28] and number of aligned reads were counted on putative transcripts.

### Transcriptome assembly

Tagcleaner v. 0.12 [29] was used to remove 454 tags. Illumina headers were converted to pre-CASAVA 1.8 version headers ending in “/1” or “/2” so that pairs could be maintained after cleaning. All reads were stringently cleaned to remove tags, ambiguous bases, duplicates, polyA/T/N tails, and low quality bases using Prinseq v.0.20.3 [30]. For the assembly of 454 reads, the miraEST v3.4.1.1 assembler [31] was used. Illumina reads were assembled with multiple odd values of *k* (23-61) using Velvet v1.2.08 and Oases v0.2.08 [32]. The single-*k-*mer Illumina assemblies were merged with a *k-*mer value of 27. The resultant Illumina and 454 assemblies were merged with miraEST v3.4.11 [31] to produce the final merged transcriptome. Additional file 1: Figure S1 depicts the workflow for the transcriptome pipeline.

Assemblies were evaluated on the basis of N25, N50, N75, cumulative length of contigs, and number of contigs. BLASTX, NCBI BLAST+ v2.2.28 [33], was run to search the *Andropogon* contigs for putative homologs to *S. bicolor* Phytozome v9.0 proteins and the NCBI nr protein database. The custom scripts Blastx.pl v 1.0 and FindFailed.pl were used to run BLASTX against the NCBI nr protein database. Significant BLASTX hits (e-value < 1e-10) were retained. Ortholog Hit Ratio (OHR) [34] was calculated for the assemblies. OHR is the length of the BLASTX hit region (the putative coding divided by three) divided by the length of the protein in the *S. bicolor* database. Therefore, OHR is an estimate of the percent of the full length protein sequence represented in the assembly. An OHR of 1 indicates a potential full length transcript. In an effort to reduce the influence of redundant or fragmented contigs, the script UniqueBlast.pl [34] was used to identify the longest contig with a significant hit to any single protein in the *S. bicolor* database. Only these contigs were used to calculate OHR.

Results of the transcriptome assembly follow. Cumulative length of sequences (Additional file 1: Figure S2), N-values (Additional file 1: Figure S3), and OHR (Additional file 1: Figure S4) values suggest that the merged assembly is more complete than the single *k*-mer assemblies and the 454 assembly. Additionally, N-values (Additional file 1: Figure S3) and OHR (Additional file 1: Figure S4) compare favorably to other similar *de novo* transcriptome assemblies. All N-values (N25, N50 and N75) are higher for single-*k-*mer Illumina assemblies (N50 > 1.3 kb for all single-*k-*mer Illumina assemblies, then for the Mira assembly of 454 reads (N50 ~ 0.8 kb) as shown in Additional file 1: Figure S3. All N-values were highest for the final merged assemblies indicating that the merged assembly may be more contiguous than the Illumina or 454 assemblies individually (Additional file 1: Figure S3). The final N50 of 3.2 kb is higher than recent *de novo* grass transcriptomes (wheat 1.4 kb [35], *Panicum hallii* 1.3 kb [36], Ma Bamboo 1.1 kb [37] , *Miscanthus* 0.7 kb [38]. High N-values alone may not indicate accurate assembly. However, OHR was also found to improve for the merged assembly. In the merged assembly ~56 % of the contigs with a BLASTX hit had an OHR of 0.5 or greater and ~74 % had an OHR of 0.8 or greater. This was a larger percentage of BLASTX hits than any of the single-*k-*mer Illumina assembly indicating that the contigs in the merged assembly seemed to be more complete in addition to having higher N-values than either the Illumina or the 454 assemblies considered individually. These OHR values are higher than other recent results (62 % ≥0.5 and 35 % ≥0.8 for *Daphnia pulex* [34], 64 % ≥0.5 and 35 % ≥0.9 for salt marsh beetle [39].

The cumulative assembly length and number of contigs (Additional file 1: Figure S2) when compared between all assemblies also suggest that the final assembly is of higher quality than the initial Illumina or 454 assemblies alone. For single-*k-*mer Illumina assemblies, intermediate *k-*mer values produced the longest assemblies while the total number of contigs decreased as the *k-*mer value increased. For the MIRA assembly of 454 reads the cumulative length of the assembly was ~20 Mb greater than the longest single-*k-*mer Illumina assemblies though the number of contigs was ~10 times lower than the single-*k-*mer Illumina assemblies. Overall, the number of contigs was high for both the single-*k-*mer Illumina assemblies (391,875 to 551,163 contigs) and the MIRA assembly of 454 reads (53,174). The final merged assembly had the smallest number of sequences (26,373) and the shortest contiguous length (~65 Mb). Taken with the increased N-values (Additional file 1: Figure S3) and OHR metrics (Additional file 1: Figure S4), the smaller length and number of contigs of the final merged transcriptome (Additional file 1: Figure S2) may indicate that redundant and fragmented transcripts from the initial assemblies were more completely assembled in the final merged transcriptome.

Cleaned Illumina reads were mapped to the final merged assembly using Bowtie2 v.2.1.0 [40] in the best mapping mode. The final transcriptome used for analysis contained transcripts with greater than or equal to 400 base pairs.

### RNA-seq data

The dataset used for analysis consisted of a total of 25,582 transcripts. Data are available as additional supporting files to this article.

We first defined SB-only transcripts as transcripts with expression levels present in sand bluestem and absent (i.e. read counts = 0 for all samples) in big bluestem. In turn, BB-only transcripts were defined as transcripts with expression levels present in big bluestem and absent (i.e. read counts = 0 for all samples) in sand bluestem.

For descriptive purposes, we then organized the data based on relative abundance of transcripts. In brief, transcripts were ranked from largest to smallest number of total mapped reads across all samples. We adapted the approach proposed by Bullard [17] and defined high-count transcripts as the top 3 % transcripts with the highest relative abundance, which accounted for 60 % of total read counts (Fig. 1). We also defined low-count transcripts as transcripts within the 60^th^ percentile of least relative abundance, which accounted for approximately 3 % of total read counts (Fig. 1). So defined, high-count transcripts and low-count transcripts were transcripts with at least 12,893 read counts or at most 462 read counts, respectively, across all samples in the dataset. We note that the proposed definitions of high-count and low-count transcripts are specific to our motivating problem and the corresponding structure of our data. Table 1 shows the breakdown of transcripts into high-count and low-count categories in the complete dataset (i.e. no filtering applied) and in the filtered data (see later).

### Construction of plasmode datasets

All plasmodes were generated using data from big bluestem samples only, given its benchmark status as a widely distributed dominant prairie grass. Null plasmode datasets were constructed as previously described [7]. Briefly, for each null plasmode, samples of big bluestem were randomly partitioned into two arbitrary groups. A total of 3 unique null plasmodes were created, reflecting the 3 possible unique combinations of 4 samples in groups of 2. So defined, no differential expression is to be expected between groups other than sample-to-sample variation. Thus, null plasmodes allow for evaluation of analysis models under the null hypothesis [7].

A total of 5 DE plasmodes were generated from each null plasmode for a total of 15 DE plasmodes, as previously described [7]. The proportion of differentially expressed transcripts in each DE plasmode was set at π = 0.2. We used edgeR classic [21] to obtain a list of estimated effect sizes for transcripts declared DE at FDR = 0.05. Estimates of effect sizes were sampled without replacement and added to log-transformed counts of randomly selected transcripts on all samples of one of the arbitrary groups in the null plasmode dataset, then back transformed to the count scale. As such, DE plasmodes combine random reshuffling of data with known effects estimated from real data and added to known transcripts. Thus, DE plasmodes allow for evaluation of analysis models in identifying truly DE as well as non-DE transcripts [7].

### Differential expression analyses

#### DESeq2

The R package DESeq2 [3] for which the read count *K*_*ij*_ for transcript *i* in sample *j* is described with a generalized linear model of the Negative Binomial family with logarithmic link, such that *K*_*ij*_ ~ *NB*(*mean* = *μ*_*ij*_, *dispersion* = *α*_*i*_ ) with mean *μ*_*ij*_ = *s*_*j*_*q*_*ij*_ and link function log (*q*_*ij*_) = *x*_*j*_*β*_*i*_, where *s*_*j*_ is the normalized library size for sample *j* as previously defined [22]. In turn, *α*_*i*_ is the variability between samples for transcript *i*, *x*_*j*_ contains the elements of the known design matrix for sample *j*, and *β*_*i*_ describes the corresponding coefficient for transcript *i*. Estimation of the dispersion parameter is conducted in 3 steps [3]. First, gene-wise dispersion estimates are obtained using maximization of the Cox-Reid adjusted conditional likelihood of the dispersion. Then, a dispersion trend is estimated using a Gamma-family generalized linear model regression. Last, a maximum a-posteriori dispersion estimate is obtained by shrinking the gene-wise dispersion estimates toward the overall dispersion trend using an empirical Bayes approach that enables borrowing of information across transcripts. DESeq2 further incorporates empirical Bayes shrinkage of logarithmic fold changes, thus enabling further borrowing of information and stable estimation for gene expression fold changes to count data, particularly for low-count genes [3]. More specifically, maximum-likelihood estimates of logarithmic fold changes are shrunk towards a zero-centered normal prior distribution to yield the final maximum a-posteriori estimates. The amount of shrinkage is inversely proportional to the amount of information an experiment provides for a given log fold change coefficient, so that transcripts with low estimated mean values *μ*_*ij*_ and high dispersion *α*_*i*_ in small datasets are pulled more strongly toward zero. Default DE testing on the shrunken LFCs is based on a Wald test, whereas a LRT alternative is also available [3].

#### EdgeR robust

R package edgeR robust [10] for which the read count *Y*_*ij*_ for transcript *i* in sample *j* is described with a generalized linear model of the Negative Binomial family with logarithmic link, such that *Y*_*ij*_ ~ *NB*(*mean* = *μ*_*ij*_, *dispersion* = *ϕ*_*i*_) with link function log (*μ*_*ij*_) = *Xβ*_*i*_ + log (*N*_*j*_), where *X* is the design matrix containing the covariates, *β*_*i*_ is a vector of regression parameters, *N*_*j*_ is the library size for sample *j*, and *ϕ*_*i*_ is the square of the biological coefficient of variation for transcript *i*. Dispersion parameters are estimated as follows. First, initial gene-wise dispersion is estimated using adjusted penalized likelihood. These estimates are then moderated by shrinkage towards a common dispersion estimate obtained by maximizing a common likelihood function. Shrinkage is determined by a prior degree of freedom parameter afforded to the shared likelihood and specified arbitrarily by the researcher [4]. Unless explicitly specified, the default value for the prior degrees of freedom is equal to 10 [41]. In turn, regression parameters *β*_*i*_ are estimated using maximum likelihood that incorporates working weights attached to each observation. Weights are attached to each observation so observations that deviate strongly from model fit are given a lower weight. Observations weights are defined as functions of a Pearson residual [10] that are iteratively updated during estimation. The dispersion estimation machinery also receives the same observation weights, so that the influence of outliers is dampened on both regression and dispersion estimates. Testing for DE is conducted using a LRT-based approach.

#### Specification of the shrinkage parameter for edgeR robust

As previously indicated, edgeR robust uses DF = 10 as a default to specify the amount of shrinkage applied to dispersion parameters [9]. While the default value for degrees of freedom is provided in the edgeR robust package as a “rule of thumb”, there is little guidance available to accurately inform specification of the DF parameter in a given dataset. Greater values of DF indicate greater shrinkage of tagwise dispersion estimates towards an overall dispersion parameter common to all transcripts.

We compared the performance of edgeR robust at varying DF specifications. In particular, we considered DF = 4, 10 and 50, to indicate a range of shrinkage around the default specification. Further, we considered using the classical edgeR software [21] to estimate DF using a quantile-adjusted conditional maximum likelihood [21]. Estimation of DF is facilitated by the simple design structure of our bluestem dataset, in which only 2 groups are being compared (i.e. BB vs SB) and no blocking or nesting design structure is apparent. We refer to this scenario as a DF = classic DF=$\widehat{\mathrm{D}}\mathrm{F}$_edgeR specification under edgeR robust.

#### Multiple testing adjustments

Following DE analyses based on either DESeq2 or EdgeR robust, transcripts were called DE based on a FDR = 0.05 using the Benjamini-Hochberg procedure [42].

#### Performance metrics

Table 2 defines performance metrics used to compare inferential performance of statistical methods. More specifically, we defined FPR as the number of false positives over the sum of false positives and true negatives. Power was defined as the number of true positives over the sum of true positives and false negatives. In turn, precision was the number of true positives over the sum of true positives and false positives, and it is also referred to as positive predictive value. Further, NPV was the number of true negatives over the sum of the true negatives and false negatives. Finally, accuracy was defined as the sum of true positives and true negatives over the total number of transcripts.

Performance metrics were computed on each plasmode dataset fitted with each statistical method for DE analyses. Each metric was then fitted with a general linear mixed model to compare methods for DE analysis accounting for plasmode dataset as a random blocking factor. Models were fitted using the GLIMMIX procedure of SAS (Version 9.3, SAS Institute Inc., Cary, NC). Residual assumptions were evaluated using studentized residuals. Pairwise comparisons in performance metrics between analyses methods were conducted using a Tukey-Kramer adjustment to prevent the inflation of type 1 error rate.

#### Filtering strategies

Filtering criteria are often applied to RNA-set datasets prior to DE analyses. A relatively common filtering criterion removes transcripts from a dataset if the total number of samples with mapped reads present for that transcript is smaller than the number of samples per treatment [8]. For our data, RP filtering removes a transcript if fewer than 4 samples had mapped reads present across all 8 samples. Another common filtering alternative strategy removes transcripts if two or more samples have CPM smaller than an arbitrary number [7]. For our data CPM-based filtering removed a transcript if two or more samples had less than 1 CPM for that transcript. This was analogous to removing any transcripts with fewer than 80 mapped reads across all samples. The number of transcripts remaining in the dataset after applying RP filtering or CPM filtering is shown in Table 1, along with the total number of transcripts in the unfiltered dataset, whereby all transcripts with at least one read count in any of the samples is included.

## Additional file

Additional file 1: Table S1. Number of high quality reads by ecotype and population for 454 and HiSeq platforms. **Figure S1.** Workflow diagram of transcriptome assembly pipeline. **Figure S2.** Cumulative length of sequences and number of sequences for various k-mer values, 454 data, and the combined 454 and HiSeq data. **Figure S3.** N values for various k-mers and MIRA 454 and MIRA clustered assemblies. **Figure S4.** Ortholog hit ratio for final MIRA clustered assembly. OHR is the length of the BLASTX hit region divided by the length of the protein, in our case using the S. bicolor database. OHR is an estimate of the percent of the full length protein sequence represented in the assembly. An OHR of 1 indicates a potential full length transcript. (DOCX 230 kb)

## Abbreviations

BB: Big bluestem
CPM: Counts per million
DE: Differential expression
DF: Degrees of freedom
FDR: False discovery rate
FPR: False positive rate
LFC: Logarithmic fold change
LRT: Likelihood ratio test
NPV: Negative predictive value
PPV: Positive predictive value
RNA-seq: RNA sequencing
RP: Reads present
SB: Sand bluestem
TPR: True positive rate
