# Supplementary material for: Inferential considerations for low-count RNA-seq transcripts: a case study on the dominant prairie grass Andropogon gerardii
Source: BMC Genomics. 2016 Feb 27;17:140. doi: 10.1186/s12864-016-2442-7 (PMC4769568; doi:10.1186/s12864-016-2442-7)

Additional file: Table S1. Number of high quality reads by ecotype and population for 454 and HiSeq platforms.

| Sequencing platform | Ecotype | Population | Sample ID  (biol rep id) | Number of high quality reads |
| --- | --- | --- | --- | --- |
| 454 |  |  |  |  |
|  | Sand bluestem | Arapaho | 2 | 616,333 |
|  | Big Bluestem | Saline | 3 | 534,633 |
|  |  |  | TOTAL | 1,150,966 |
| Illumina HiSeq 100PE |  |  |  |  |
|  | Sand bluestem | Arapaho | 2 | 59,344,312 |
|  | Sand bluestem | Arapaho | 4 | 43,668,902 |
|  | Sand bluestem | Arapaho | 6 | 45,661,952 |
|  | Sand bluestem | Arapaho | 8 | 54,831,738 |
|  |  |  | 4 reps | 203,506,904 |
|  | Big Bluestem | Saline | 3 | 42,340,768 |
|  | Big Bluestem | Saline | 4 | 45,036,800 |
|  | Big Bluestem | Saline | 6 | 38,049,892 |
|  | Big Bluestem | Saline | 7 | 47,092,000 |
|  |  |  | 4 reps | 172,519,460 |
|  |  |  | TOTAL 8 reps | 376,026,364 |

Additional file: Figure S1. Workflow diagram of transcriptome assembly pipeline.


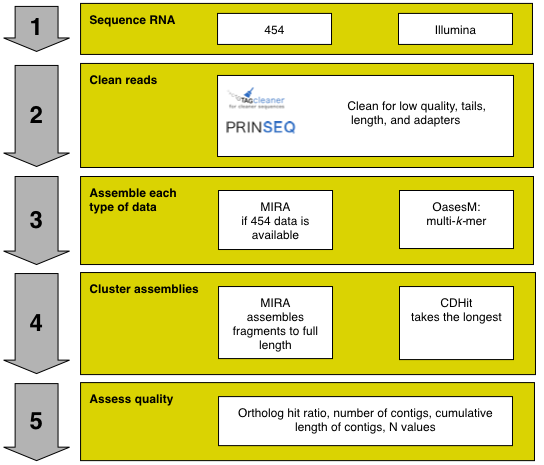


Additional file: Figure S2. Cumulative length of sequences and number of sequences for various k-mer values, 454 data, and the combined 454 and HiSeq data.


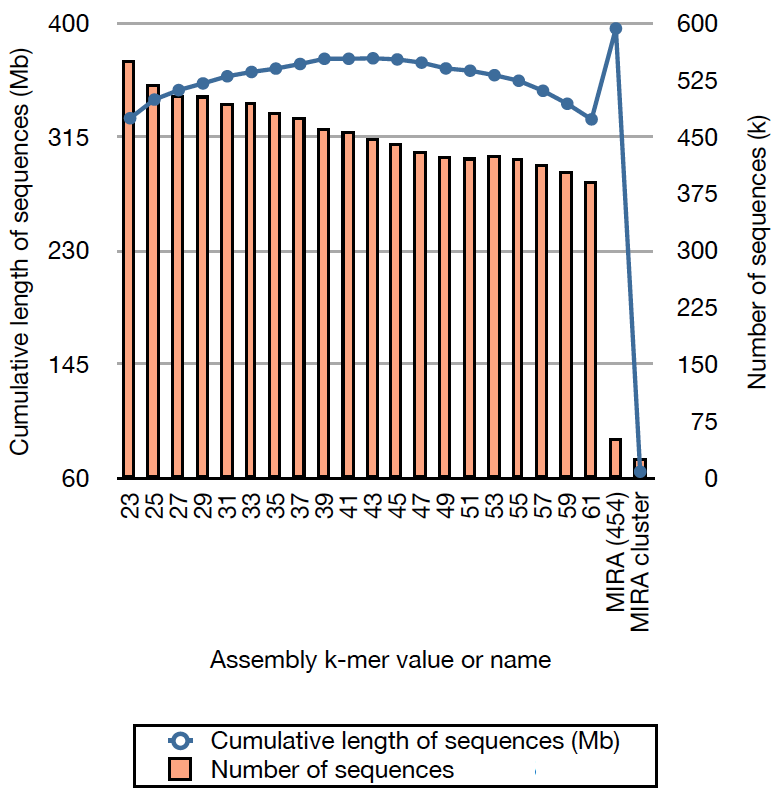


Additional file: Figure S3. N values for various k-mers and MIRA 454 and MIRA clustered assemblies.


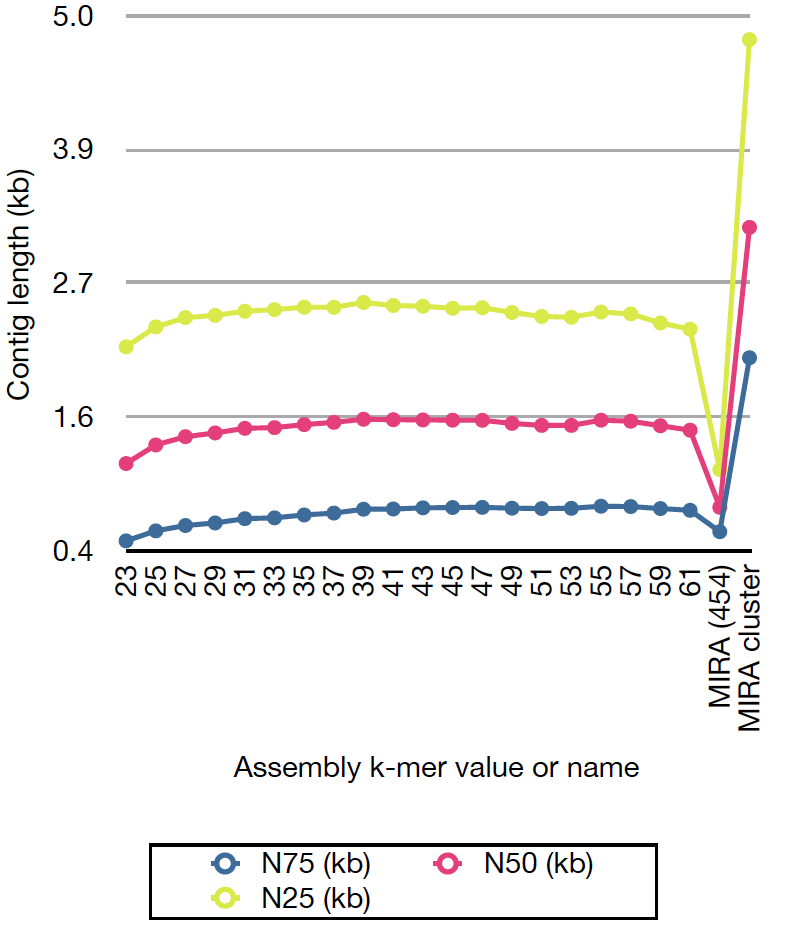


Additional file: Figure S4. Ortholog hit ratio for final MIRA clustered assembly. OHR is the length of the BLASTX hit region divided by the length of the protein, in our case using the *S. bicolor* database. OHR is an estimate of the percent of the full length protein sequence represented in the assembly. An OHR of 1 indicates a potential full length transcript.


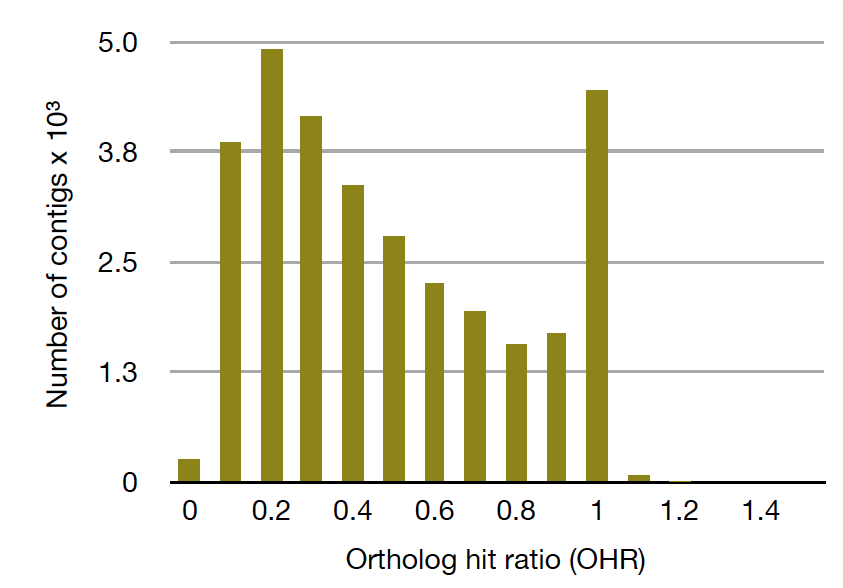

Supplement: Additional file 1: Table S1. — Number of high quality reads by ecotype and population for 454 and HiSeq platforms. Figure S1. Workflow diagram of transcriptome assembly pipeline. Figure S2. Cumulative length of sequences and number of sequences for various k-mer values, 454 data, and the combined 454 and HiSeq data. Figure S3. N values for various k-mers and MIRA 454 and MIRA clustered assemblies. Figure S4. Ortholog hit ratio for final MIRA clustered assembly. OHR is the length of the BLASTX hit region divided by the length of the protein, in our case using the S. bicolor database. OHR is an estimate of the percent of the full length protein sequence represented in the assembly. An OHR of 1 indicates a potential full length transcript. (DOCX 230 kb) [file 12864_2016_2442_MOESM1_ESM.docx]
